# Proteoglycans as Mediators of Cancer Tissue Mechanics

**DOI:** 10.3389/fcell.2020.569377

**Published:** 2020-11-30

**Authors:** Anna Barkovskaya, Alexander Buffone, Martin Žídek, Valerie M. Weaver

**Affiliations:** ^1^Center for Bioengineering & Tissue Regeneration, Department of Surgery, University of California, San Francisco, San Francisco, CA, United States; ^2^Department of Bioengineering, University of Pennsylvania, Philadelphia, PA, United States; ^3^Department of Radiation Oncology, Eli and Edythe Broad Center of Regeneration Medicine and Stem Cell Research, University of California, San Francisco, San Francisco, CA, United States; ^4^Department of Bioengineering, Eli and Edythe Broad Center of Regeneration Medicine and Stem Cell Research, University of California, San Francisco, San Francisco, CA, United States; ^5^Department of Therapeutic Sciences, Eli and Edythe Broad Center of Regeneration Medicine and Stem Cell Research, University of California, San Francisco, San Francisco, CA, United States; ^6^UCSF Helen Diller Family Comprehensive Cancer Center, University of California, San Francisco, San Francisco, CA, United States

**Keywords:** proteoglycans, GAG, cancer, mechanosignaling, glycocalyx

## Abstract

Proteoglycans are a diverse group of molecules which are characterized by a central protein backbone that is decorated with a variety of linear sulfated glycosaminoglycan side chains. Proteoglycans contribute significantly to the biochemical and mechanical properties of the interstitial extracellular matrix where they modulate cellular behavior by engaging transmembrane receptors. Proteoglycans also comprise a major component of the cellular glycocalyx to influence transmembrane receptor structure/function and mechanosignaling. Through their ability to initiate biochemical and mechanosignaling in cells, proteoglycans elicit profound effects on proliferation, adhesion and migration. Pathologies including cancer and cardiovascular disease are characterized by perturbed expression of proteoglycans where they compromise cell and tissue behavior by stiffening the extracellular matrix and increasing the bulkiness of the glycocalyx. Increasing evidence indicates that a bulky glycocalyx and proteoglycan-enriched extracellular matrix promote malignant transformation, increase cancer aggression and alter anti-tumor therapy response. In this review, we focus on the contribution of proteoglycans to mechanobiology in the context of normal and transformed tissues. We discuss the significance of proteoglycans for therapy response, and the current experimental strategies that target proteoglycans to sensitize cancer cells to treatment.

## Introduction

Proteoglycans are proteins with covalently attached GAG chains, which regulate tissue development and have been implicated in pathologies such as cancer. Proteoglycans engage cell surface receptors to induce biochemical signaling, and help modulate the mechanical properties of the ECM including its stiffness. Alongside glycoproteins and glycolipids, proteoglycans act as structural components of the cellular glycocalyx, where they influence cell signaling by regulating the structure function of transmembrane receptors. Here we focus on the role of proteoglycans in tumor mechanics including their impact on extracellular matrix stiffness and the tumor glycocalyx.

## Mechano-Sensing in Cancer

In cancer development, oncogenic mutations and the loss of tumor suppressors have long been viewed as the critical initiating events. Nevertheless, there is a growing consensus that even the cells carrying the most powerful oncogenic mutations require an intricate set of microenvironmental conditions to foster their malignant transformation and cancer progression. Among these microenvironmental factors, tissue stiffening which accompanies the fibrotic response that characterizes all solid tumors, has emerged as a key factor that can foster malignancy and collaborate with oncogenes to disrupt tissue organization and promote the growth, survival, invasion and ultimately the metastatic dissemination of the cancer cells (Northey et al., 2017). Maintaining tissue organization requires retention of a state of tensional homeostasis that is mediated by a balance between the stiffness of the tissue stroma and the actomyosin contractility of the cells within the tissue. An abnormal and sustained increase in the stiffness of the stroma such as occurs with fibrosis will increase the contractility of the cells in the tissue to destabilize cell-cell junctions, compromise polarity and destroy tissue architecture. Similarly, an increase in cellular contractility, that is induced following oncogene activation (e.g., Ras or Her2), disrupts tissue architecture as well. It destabilizes cell-cell adhesions, and stimulates the remodeling and stiffening of the ECM until such time as the cell reaches a state of tensional equilibrium with its mechanical microenvironment (Paszek et al., 2005). Thus, chronically elevated tension will compromise tissue architecture and this in turn can foster malignant transformation and promote tumor progression (DuFort et al., 2011; Samuel et al., 2011).

A stiffened tissue is frequently accompanied by chronic inflammation which, in turn, increases stiffness even further (Maller et al., 2020). Consistently, pathologies such as kidney, lung and liver fibrosis are characterized by inflammation as well as increased levels of ECM proteins including interstitial collagen, that stiffen the stroma of the tissue. Tissue fibrosis is accompanied by increased risk of cancer development (Adler et al., 2016; Noguchi et al., 2018). Moreover, tissue stiffening correlates clinically with tumor progression and aggression in malignant gliomas (Miroshnikova et al., 2016; Barnes et al., 2018), as well as adenocarcinoma of the breast (Acerbi et al., 2015), and pancreas (Laklai et al., 2016).

## Mechanical Stress and Mechano-Transduction

**FIGURE 1:**
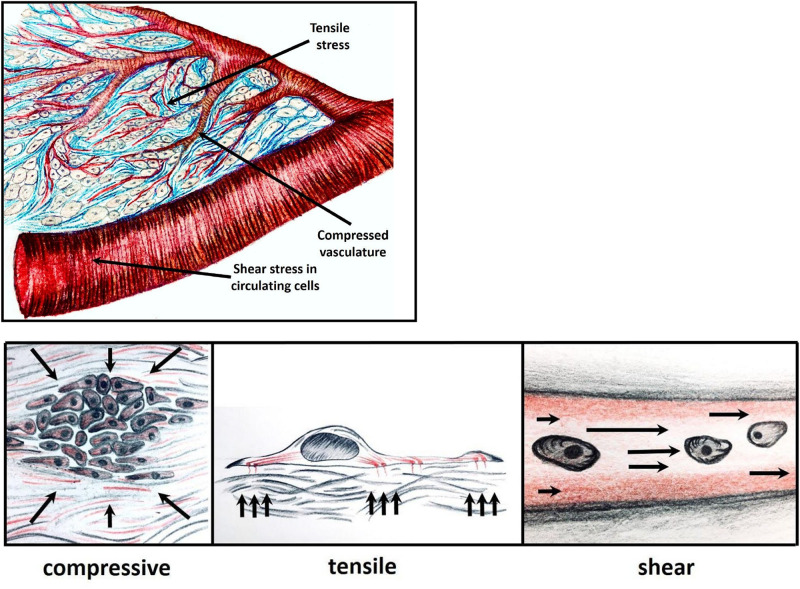
Within a growing tumor, cells are subjected to mechanical forces, in response to which the cells exert tensile, compressive or shear stress. Tensile pressure (tissue tension), stems from ECM stiffening and remodeling; compressive pressure applied occurs when tumor cells proliferate in a confined space leading to vascular compression and damage; and shear force that is experienced by the cancer cells in the circulation.

There are three types of mechanical stress that tumor cells experience: tensile, compressive and shear pressure, reviewed in (Butcher et al., 2009; Northey et al., 2017) (Figure 1).

### Tensile Stress

Tensile stress, or tension, arises from an increase in the local tissue stiffness caused by a rigid ECM and its ability to stimulate actomyosin contractility in the cells within the tissue. This is associated with ECM remodeling, accumulation of proteins such as fibronectin and increased abundance and cross-linking of collagen fibers (Levental et al., 2009; Schedin and Keely, 2011). While type I collagen is critical for increasing the tensile strength of most peripheral tissues, hyaluronic acid, tenascin and small proteoglycans including biglycan and lumican, contribute substantially to the stiffness of the brain parenchyma (Kim and Kumar, 2014; Miroshnikova et al., 2016).

Cellular tension can also arise through the increased activity of GTPases such as Rho that are stimulated by elevated cytokine activation of G protein coupled receptors or by activated oncogenes such as Ras (Samuel et al., 2011; Sanz-Moreno et al., 2011; Laklai et al., 2016). High mechanical tension is sensed by cellular transmembrane receptors such as integrins. Integrins are hetero-dimeric transmembrane proteins that serve as primary receptors of ECM components. Integrins consist of dimeric α and β chains that each have multiple isoforms, creating 24 known unique combinations that are expressed in a tissue-specific way and selectively bind to the ECM proteins (Humphries et al., 2006). Integrin activation can be induced either outside – in via ligand binding or inside - out via intracellular signaling. In either instance, integrin activation induces a conformational change in the heterodimer that unfolds the integrin to potentiate its ligand binding and facilitate recruitment of cytoplasmic proteins that initiate integrin clustering to form nascent adhesions.

**FIGURE 2:**
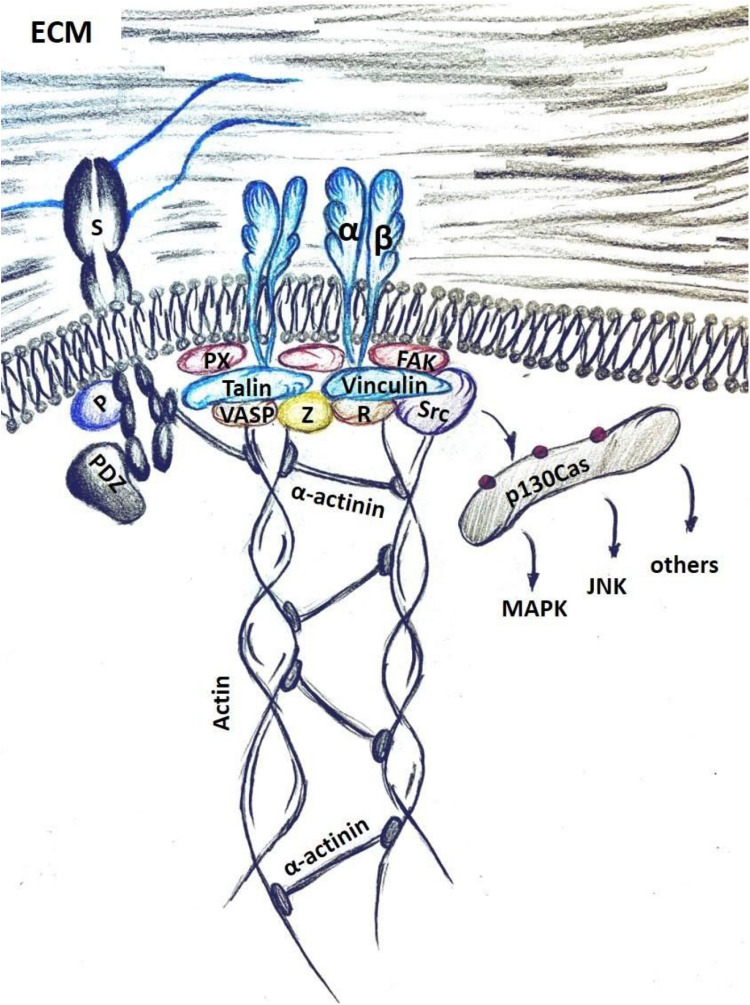
Mechano-transduction in cells is mediated through integrin activation upon binding to the ECM components, such as fibronectin. Syndecans (S) facilitate this interaction by binding the ECM with HS chains. Intracellular syndecan domains activate signaling with PKCα (P) and via binding to PDZ-motif proteins. Integrin activation recruits a host of focal adhesion components, including integrin-binding adaptor proteins – talin and paxillin (PX); actin binding cytoskeletal proteins VASP and vinculin (V); and focal-adhesion associated kinases Src, FAK and ROCK. VASP and zyxin (Z) facilitate actin polymerization and connect it to the focal adhesion complex. Phosphorylation of p130Cas by Src allows docking and activation of several signaling pathways, such as the MAPK, the c-Jun N-terminal kinase (JNK) pathway and others.

Focal adhesion formation, however, requires cellular actomyosin contractility to reach a sufficient level that is proportional to the stiffness of the ECM. At a critical threshold talin-vinculin binding is favored as is the recruitment and activation of focal adhesion kinase (FAK), ROCKI and ROCKII, Src, as well as several adaptor proteins, including paxillin that foster the assembly of focal adhesions. Within these adhesion complexes vinculin and talin function as “molecular clutches” linking the integrin directly to the network of actin filaments underneath the cell membrane. When cellular tension is sufficiently high and prolonged, these molecular clutches will promote the formation of stress fibers which will then be further strengthened and stabilized with participation of the final components of the focal adhesion complex – zyxin and VASP (Kanchanawong et al., 2010) (Figure 2).

In response to focal adhesion assembly, FAK and Src kinases, phosphorylate a large scaffolding protein p130Cas. p130Cas acts as a docking block for multiple signaling proteins that can activate each other in close proximity, therefore amplifying their down-stream signaling cascades. These include ILK – CDC42 and ILK – Rac pathways, which regulate filopodia and invadopodia formation and cell polarization and contractility (Alexander et al., 2008). These functions allow the cells to initiate movement in response to mechano-stimulation, or, in the case of cancer cells, to move toward and invade the neighboring tissues after being exposed to a stiff environment.

In addition, cell survival and proliferative pathways are also induced via interaction with p130Cas. Erk and PI3K signaling is up-regulated upon integrin activation in a p130Cas-dependent manner (Cabodi et al., 2010; Janoštiak et al., 2014). Further evidence of p130Cas involvement in mechanosignaling comes from a study which determined that physical stretching of the cells on elastic substrates induced p130Cas to assume an unfolded conformation, open to phosphorylation by Src. This, in turn, promoted down-stream signaling. Notably, p130Cas unfolding and phosphorylation is significantly higher among the cells on the edges of the 3D *in vitro* colonies, compared to the bulk of the cells inside. This suggests that mechanical stretching directly activates pro-survival signaling in invading cells enabling them to migrate and disseminate (Sawada et al., 2006).

Elevated cellular contractility is also characteristic of the cells that carry a bulky glycocalyx. Glycocalyx is a dense cell surface coating composed of glycoproteins and proteoglycans, which reinforce the external barrier of a cell, and actively regulate mechano-transduction and growth factor signaling. In aggressive metastatic cancer cells, glycocalyx is frequently enhanced (Paszek et al., 2014; Barnes et al., 2018). Bulky glycocalyx was shown to facilitate adhesion assembly and augment integrin-mediated signaling (Paszek et al., 2014).

Finally, stiff microenvironment activates a critical mechanosignaling pathway –YAP and transcriptional coactivator with a PDZ-binding motif (TAZ) (Dupont et al., 2011). YAP/TAZ are transcriptional co-activators that shuttle between cytoplasm and the nucleus, where they bind to DNA transcription factor TEAD and activate expression of its target genes, supporting cell survival and decreasing apoptosis. This signaling pathway is mechano-responsive, and several mechanisms for mechano-regulation of YAP/TAZ have been proposed. First, inhibition of ROCK kinase prevents YAP/TAZ nuclear localization (Dupont et al., 2011), which suggests that focal adhesion assembly is required for YAP/TAZ signaling. In addition, inhibitors of actomyosin and actin polymerization, as well as integrins, also inhibit YAP/TAZ (Dupont, 2016). Finally, it has also been shown that in cells stretched out on a stiff substrate, the nucleus is compressed. This causes YAP/TAZ to translocate directly inside via nuclear import channels, bypassing up-stream regulation, and to induce expression of its target genes (Elosegui-Artola et al., 2017). These results point to YAP/TAZ activation as a sensor of mechanosignaling.

Several approaches have been developed to explore the causal relationship between tissue tension and disease development including malignant transformation and tumor progression. Cellular mechanosignaling and actomyosin actomyosin tension reduction can be achieved by inhibiting integrin focal adhesion signaling or integrin focal adhesion assembly via knockdown or inhibition of key adhesion components including talin, vinculin, focal adhesion kinase and ROCK. Mechanosignaling and actomyosin tension can be enhanced through expression of a β1-integrin engineered to promote inter-molecular associations that foster clustering of the molecules by introducing a mutant β1-integrin with a single amino acid substitution: V737N. This single substitution of hydrophobic valine residue with a hydrophilic asparagine promotes integrin clustering by reducing repulsive forces in the transmembrane domain of the integrin. Expression of the V737N β1-integrin enhances the assembly of focal adhesions accompanied by elevated p397FAK and increased ROCK activity that translate into higher actomyosin contractility and potentiate growth factor receptor dependent activation of MAPK, PI3K and Stat3 signaling (Paszek et al., 2005; Levental et al., 2009; Laklai et al., 2016). Conversely, reducing tenascin C expression in aggressive glioblastoma cells significantly decreases the stiffness of the brain tumor ECM leading to significantly lower tumor aggression (Miroshnikova et al., 2016).

In matrix collagen-rich tissues, higher stiffness coupled with the reorganization of the collagen into thickened, oriented fibers facilitates the directed invasion of the cancer cells to promote their migration through the interstitial stroma that ultimately favors their dissemination and metastasis (Ahmadzadeh et al., 2017). Cells exposed to a chronically stiffened ECM with sustained myc, catenin, YAP/TAZ and TGFβ activity often undergo an EMT that promotes their phenotypic switch to a motile state that is highly resistant to anti-cancer treatments (Barnes et al., 2018). Epithelial tumor cells that have undergone an EMT down-regulate cell-cell adhesion receptors such as E-cadherin that compromise their potential to maintain polarized tissue structures. In addition to the loss of E-cadherin, polarization is also accompanied by a decrease in syndecan-1 proteoglycan on the cell surface (Sun et al., 1998). Syndecan-1 loss was later found to independently induce the EMT in several cancers, leading to increased migration and invasion (Wang et al., 2018). Cells that have undergone EMT are also more contractile and exert higher forces at their integrin adhesions (Mekhdjian et al., 2017), decrease cytokeratin expression and upregulate the intermediate filament protein vimentin and exhibit a softening of their nuclei (Liu et al., 2015; Northey et al., 2017). Intriguingly, EMT substantially increases expression of glycoproteins that contribute to the cellular glycocalyx. Cells with a bulky glycocalyx can relax the bulk of their cortical tension and this allows them to squeeze through the confined spaces often encountered within a rigid, dense tumor microenvironment (Shurer et al., 2019). Consistently, tumors that express a bulkier glycocalyx are often more aggressive and metastatic and circulating tumor cells frequently express high levels of glycoproteins that contribute to a bulky glycocalyx (Paszek et al., 2014).

Not surprisingly, the stiffened, high-tension tumor environment stimulates growth factor and cytokine dependent cancer cell growth and survival, and promotes invasion, migration and dissemination of the tumor cells. Consistently, breast and pancreatic tumor cells and glioblastoma cells in which integrin-dependent mechanosignaling was potentiated through expression of the V737N mutant β1-integrin, undergo an EMT that fosters their motility and invasion in culture and *in vivo*, and reduces the survival of experimentally manipulated mice (Laklai et al., 2016; Mekhdjian et al., 2017; Barnes et al., 2018).

### Compression Stress

The rapid proliferation of tumor cells within a confined area can increase compression stress. Compression stress in tumors can also arise through elevated water retention mediated by increased concentration of proteoglycans and hyaluronic acid (DuFort et al., 2016). Compression stress within a confined tumor can create a mechanical stress gradient with the forces declining at the tumor periphery. High compression stress within the tumor can occlude blood and lymphatic vessel integrity that induces tumor hypoxia and increases interstitial fluid pressure (Sarntinoranont et al., 2003). High tumor compression stress contributes to cancer aggression either by directly activating signaling pathways or by indirectly inducing hypoxia to stimulate HIF1a-dependent gene expression that each can increase tumor cell growth, survival and invasion, and promote EMT to drive cancer aggression and metastasis (Tse et al., 2012).

### Shear Stress

Shear stress is yet another type of mechanical stress cancer cells experience in the tumor tissue. The shear stress is created by the blood and interstitial fluid flow the cancer cells encounter once they extravasate into the vasculature or lymphatics during their metastatic dissemination. These shear stresses can stimulate signaling pathways that enhance tumor cell aggression and influence vessel patency. For instance, Polacheck et al. (2017) show how vascular flow can induce cleavage of the transmembrane Notch 1 receptor that permits the resulting Notch transmembrane domain to form a complex with adherens junctions that enhanced cell-cell adhesion stability. In cancer cells, continuous exposure to fluid flow promotes migration and invasion capacity. Some reports suggest that proteoglycan-rich glycocalyx acts as a mechano-sensor of the interstitial flow and enables mechanosignaling activation, while enzymatic reduction of glycocalyx bulk inhibits flow-induced signaling (Shi et al., 2011; Qazi et al., 2013).

In summary, as structural components of the glycocalyx and the ECM, proteoglycans function as integral regulators of bio-mechanics in both normal and malignant tissues. The following sections provide an overview of the proteoglycan diversity, describe their known function in cancer development, and summarize the reports of proteoglycans’ involvement in the regulation of mechanosignaling.

## Glycosaminoglycan (GAG) Attachments to Proteoglycans

Proteoglycans are a class of glycosylated proteins widely expressed in various tissues, which play an important role in a variety of cellular interactions and signaling events (Couchman and Pataki, 2012). Proteoglycans are one of the principal components of the mammalian glycocalyx, which along with glycoproteins and glycolipids form the sponge-like mix of proteins and lipids which acts as a barrier between the cell surface and the extracellular matrix (ECM), and regulates the inter actions between the two (Monne et al., 2013; Buffone and Weaver, 2020). In addition, proteoglycans are a major component of the ECM and are also present intracellularly and pericellularly. Proteoglycans are characterized by at least one covalently bound GAG chain, although they can also contain other N-, and O-linked glycans found in glycoproteins or glycolipids (Mulloy and Rider, 2006; Nikitovic et al., 2018). These GAG chains have very distinct glycosylation patterns which consist of repeating disaccharide units, are long (∼80 monosaccharides for GAGs while ∼10–15 for *N*-glycans), are sulfated at various different points of the GAG chain and of each monosaccharide, and have a much more linear structure as compared to *N*- or *O*-glycans (Iozzo and Schaefer, 2015; Lindahl et al., 2015). Furthermore, GAG chains are characterized by the Galactose–Galactose–Xylose motif (Gal–Gal–Xyl), which initiates the chains and can contain either IdoA or GlcA, monosaccharides unique to GAG (Gandhi and Mancera, 2008; Pomin and Mulloy, 2018). Furthermore, GAGs differ from glycoproteins and glycolipids in that the GlcA/IdoA and hexosamine sugars form repeating disaccharides which compose the long GAG chains.

There are five main GAG chains: heparin, CS, DS, KS, and HS, all of which are sulfated (Soares da Costa et al., 2017). Each GAG consists of unique monosaccharide building blocks as: heparin contains GlcNAc, GalNAc, IdoA, and GlcA (Shriver et al., 2012), CS contains GalNAc and GlcA, DS contains IdoA and GalNAc, KS contains galactose (Gal) and GlcNAc (Funderburgh, 2002), and HS contains GlcNAc, GalNAc, IdoA, and GlcA (Lindahl et al., 2015). The differences in composition of each of the GAGs, which have been determined by sequencing for most of the GAGs except for KS, expressed on proteoglycans have profound effects on their function both inside and outside of the cell.

## Cellular Localization of Proteoglycans

**FIGURE 3:**
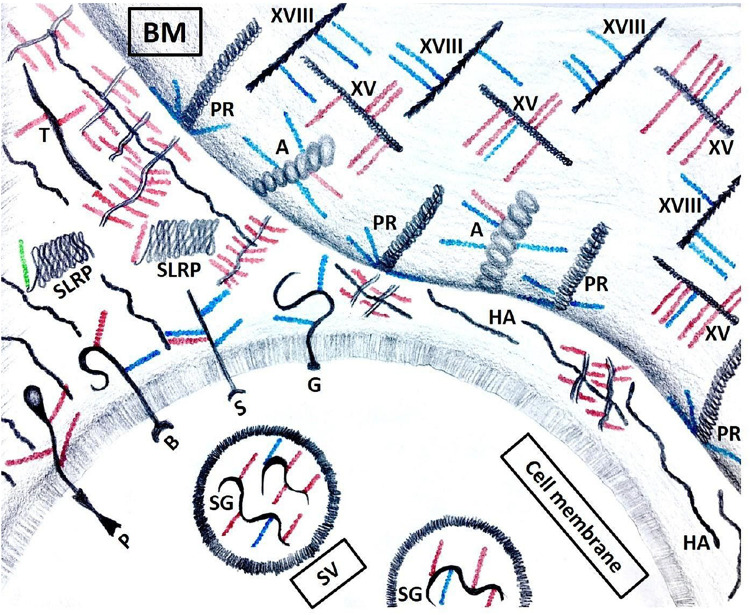
Proteoglycans’ localization. Serglycin (SG) is the only intracellular proteoglycan, found in secretory vesicles (SV). It carries both HS (blue) and CS (red) GAG chains. Transmembrane proteoglycans include, but are not limited to phosphacan (P), betaglycan (B), syndecans (S) and glypicans (G). In the ECM, SLRPs are short proteoglycans that carry either a CS or a DS (green) GAG chain. Other proteoglycans include testican (T) and hyalectins that aggregate with hyaluronic acid (HA). Basement membrane (BM) contains agrin (A), perlecan (PR) and collagens XV (XV) and XVIII (XVIII).

An important distinction between proteoglycans and other glycosylated molecules such as mucins, glycoproteins, and glycolipids, which are primarily present on the cell surface (Buffone et al., 2013, 2017; Mondal et al., 2016), is that proteoglycans can be localized either intracellularly, pericellularly, on the cell surface, or be shed into the extracellular matrix (Figure 3) (Vogel and Peterson, 1981; Yanagishita, 1993; Lin, 2004; Iozzo et al., 2009). The different members of the proteoglycan classes and their functions have been beautifully reviewed by others (Iozzo and Schaefer, 2015), but we will briefly cover the pertinent proteoglycans in terms of the topic of this review.

### Intracellular Proteoglycans

There is only one proteoglycan in the mammalian genome which resides in the intracellular space and that is serglycin. Serglycin is interesting as it is located in the secretory granules of cells and holds together all of the proteases and other components of the granule (Kolset and Tveit, 2008). Serglycin can carry a variable number of heparin or CS side chains (Nugent, 2000).

### Cell-Surface Proteoglycans

There are two major families of cell-surface proteoglycans: syndecans 1–4 (Cheng et al., 2016) which have a transmembrane domain and glypicans 1–6 (Li N. et al., 2018) which are anchored to the cell membrane through a GPI lipid. Syndecans have primarily HS GAGs but can also have CS chains which are implicated in a variety of biological processes, most critically in binding growth factors in tissues (Forsten-Williams et al., 2008). Enzymatic processing of syndecans represents an important regulator of their function. Syndecans can be secreted into the pericellular environment by MMPs (Rossi et al., 2014) and into the extracellular matrix through partial degradation of their domains by proteases to perform other functions (Teng et al., 2012).

Heparanase is an endoglycosidase that can cleave heparan sulfate side chains. This can result in the release of growth factors or chemokines bound by syndecans. In many malignancies, high expression of heparanase has been associated with an aggressive tumor phenotype (Ramani et al., 2013). Furthermore, post-assembly processing by plasma membrane-bound endo-6-*O*-sulfatases (SULF1 and SULF2) changes the sulfation pattern of HS chains, which in turn leads to an altered ligand/receptor interaction (Bishop et al., 2007). In liver cancer, the activation of SULF1/SULF2 promotes the tumor growth (Graham et al., 2016).

Glypicans are HSPGs, that are linked to the cell surface by GPI anchor. Glypicans can modulate a variety of cell signaling pathways including Wnt, frizzled, BMP, hedgehog (Hh), and bFGF (Cassiman et al., 1992; Capurro et al., 2005, 2008; Filmus and Capurro, 2014). Increased expression of glypicans has been found in pediatric cancers and liver cancer (Li N. et al., 2018). Other proteoglycans which reside at the cell surface include betaglycan (Bilandzic and Stenvers, 2012) and phosphacan (Maurel et al., 1994). They are discussed further in other reviews (Iozzo and Schaefer, 2015).

### Pericellular Proteoglycans

There are four pericellular proteoglycans that reside predominantly within the basement membrane: perlecan, agrin, and collagens XV and XVIII (Iozzo et al., 2009). Perlecan is a massive proteoglycan (>500 KD) which contains HS GAG chains that cross-link cell surface molecules to ECM components in the basement membrane (Gubbiotti et al., 2017). Perlecan is secreted by smooth muscle and vascular cells and plays a role in a variety of cellular processes including cell adhesion, inflammation, wound healing, endocytosis, and cardiovascular development (Whitelock et al., 1999; Sasse et al., 2008; Zoeller et al., 2008; Jung et al., 2013). Agrin is a similar proteoglycan in structure to perlecan that also cross-links the cell surface to the ECM that differs from perlecan by having both HS and CS GAGs and is expressed primarily in the brain where it is known to interact with NCAMs (Tsen et al., 1995; Winzen et al., 2003). Finally, collagen XV, which carries CS GAG chains, and collagen XVIII, with HS chains, are proteoglycans which are present in all human and mouse basement membranes that play distinct roles in the structural support and angiogenesis (Heinämäki et al., 1994; Eklund et al., 2001).

### Extracellular Matrix Proteoglycans

The ECM proteoglycans are the largest class of proteoglycans. This group of molecules consists of the calcium binding testican family, the SLRPs and lectin (hyalectin). Hyaluronic acid is also frequently present in the extracellular space. Testican proteoglycans carry HS and CS GAGs (Bonnet et al., 1992), are modular in structure like perlecan, and are found in both the testis and CNS where they play a strong role in neuronal development (Schnepp et al., 2005). Conversely, SLRPs contain over 18 distinct members and are much smaller than other proteoglycans (30–40 kDa). They are defined by a core that consists of leucine-rich repeats (Iozzo et al., 2011). SLRPs are widely present in almost all of the ECM in the meninges, pericardium and peritoneum and are involved in regulating organ shape (Iozzo, 1997, 1999).

The hyalectin class of proteoglycans has four members: aggrecan, versican, neurocan, and brevican, which are characterized by tri-domain structure in which the N-terminus binds hyaluronan, the C-terminus binds lectins, and the central domain contains the GAG (predominantly CS) side chains (Iozzo, 1998). The hyalectins play important roles in maintaining the structure of the brain, cartilage, and vasculature. Aggrecan is known to form large complexes (∼200 MDa) with hyaluronan, collagen, and other proteoglycans and glycoproteins and this hydrated gel-like ECM structure forms the load bearing component of cartilage (Heinegård, 2009; Wight et al., 2011). Versican has four isoforms (V0, V1, V2, and V3) which are differentially expressed in tissues such as the brain, heart, and vascular smooth muscle (Zimmermann and Ruoslahti, 1989; Naso et al., 1994; Zimmermann and Dours-Zimmermann, 2008). Versican’s function is to facilitate cell migration and resolution of inflammation as it interacts with cell surface receptors on the leukocyte surface to enhance interactions with the vasculature (Wight et al., 2014). Neurocan is a proteoglycan with CS GAGs expressed in the brain that binds to NCAM, tenascin, and hyaluronan and inhibits neurite outgrowth at the sites of injury (Davies et al., 2004; Liu et al., 2006). Finally, brevican is a critical proteoglycan in brain function as its expression has been linked to glioblastoma, brain tissue injury, and Alzheimer’s disease (Yamada et al., 1994; Frischknecht and Seidenbecher, 2012).

## Proteoglycans in Cancer

Several distinct functions of proteoglycans define their complex role in biological systems. To begin with, proteoglycans promote cell-to-cell and cell-to-matrix interactions. Proteoglycans also trap and release various growth factors and cytokines. For example, HS GAG chains of transmembrane proteoglycans can bind bFGF (Faham et al., 1996; Li Y. et al., 2016). As bFGF and other growth factors trapped by the GAG chains of proteoglycans are gradually dissolved, they prompt proliferation of the neighboring cells, and induce vascular growth and tissue regeneration (Hao et al., 2018; Li R. et al., 2018). In addition, enzymatic processing of proteoglycans by proteases and heparanases represents another important factor that modifies their biological activity (Cheng et al., 2016).

Proteoglycans, most prominently HSPGs, are often dysregulated during tumor development (Fjeldstad and Kolset, 2005; Varki et al., 2016). However, their role in cancer is highly context dependent. The following section describes the known contribution of intracellular, pericellular and ECM proteoglycans in cancer development and progression.

### Intracellular

Heparin- and CS-decorated serglycin is the sole known intracellular proteoglycan, expressed most prominently by the endothelial, hematopoietic and myeloid cells. It can be detected both intracellularly, where it localizes to the secretory vesicles, and is secreted into the extracellular space (Schick et al., 2001; Kolseth et al., 2015). Serglycin secreted by both cancer-associated stroma cells, as well as the cancer cells themselves, binds to the transmembrane glycoprotein CD44 (Guo et al., 2017).

Activation of CD44 in cancer cells promotes stemness, drives sphere formation, and increases invasion and migration (Skandalis et al., 2019). These effects are mediated through a transcription factor, Nanog, which sustains the non-committed state of the cells (Guo et al., 2017). Completing a positive-feedback loop, serglycin itself appears to promote CD44 expression, since a knock-down of serglycin leads to a drop in the cells with high CD44 levels (Zhang et al., 2020). In lung and colorectal cancer, high expression and secretion of serglycin is associated with a poor clinical outlook and treatment resistance (Xu et al., 2018), while the knock-down of serglycin decreases tumor burden *in vivo* (Guo et al., 2017). In line with these findings, serglycin was also found to support stemness in glioblastoma cells, where it prevents astrocytic differentiation and promotes cell proliferation and tumor growth (Manou et al., 2020).

### Pericellular

The clinical consequence of high expression of transmembrane proteoglycans is variable among cancer patients. The current consensus emphasizes a highly context- and cancer type-dependent manner in which certain proteoglycans regulate the tumor development and progression.

#### Syndecan-1 (Sdc-1)

Sdc-1 is found in several normal tissues, as well as in the malignant tumors (Kind et al., 2019). In some cases, high expression of Sdc-1 has been linked to poor prognosis. Notably, high levels of Sdc-1 in breast cancer cells correlated with higher incidence of brain metastasis (Sayyad et al., 2019). Sdc-1 is also more abundant in the ER-negative tumors, suggesting that its expression marks this more aggressive breast cancer phenotype (Barbareschi et al., 2003; Baba et al., 2006; Qiao et al., 2019). This has been corroborated by reports showing that Sdc-1 expression is directly repressed by ER (Fleurot et al., 2019). In agreement with this, recent work described Sdc-1 as an important marker of CSC phenotype in inflammatory breast cancer – a subtype with a notoriously poor prognosis (Ibrahim et al., 2017). In that study, Stat3, NFκB, Notch-1, Notch-2, and EGFR signaling was significantly reduced upon Sdc-1 knock-down. These data suggest that Sdc-1 could serve as a modulator of CSC phenotype and may be a promising therapeutic target. Notch pathway activation may specifically rely on the HS GAG chains of the Sdc-1, as a recent study showed that overexpression of HS-sulfotransferase enzymes regulates Notch signaling in breast cancer cells. However, this effect is specific to the triple-negative breast cancer (TNBC) (Teixeira et al., 2020).

These findings are in contrast with other work, where Sdc-1 depletion led to the increased breast cancer cell motility and invasion facilitated by stronger integrin adhesion to ECM components, such as fibronectin (Ibrahim et al., 2012; Hassan et al., 2013). Some of this discrepancy in the role of Sdc-1 in breast cancer development may also be due to its differential expression between cell types. For example, one study of the normal breast tissue found that Sdc-1 expression was higher in the stromal component compared to lobular epithelium (Lundström et al., 2006). The authors found that in women with high mammographic density (MD), Sdc-1 expression shifted from epithelial to stromal cells. As high MD has been linked to increased breast cancer incidence (Vachon et al., 2007), this suggests that stromal Sdc-1 expression is important for breast cancer development. In support of this, orthotopic injection of aggressive murine mammary tumor cell lines into Sdc-1–/– mice prevented lung metastasis compared to wild-type animals. These findings identify Sdc-1 as a key component of a lung metastatic niche (Chute et al., 2018).

Pro- and anti-tumorigenic role of Sdc-1 may also be context-dependent. In colorectal cancer cells, high expression of Sdc-1 reduced tumor growth and invasion through down-regulation of MAPK and Stat3 signaling (Wang et al., 2019). Additionally, low expression of Sdc-1 corresponded with poor prognosis in colorectal cancer patients (Li K. et al., 2019). In this cancer type, Sdc-1 silencing promoted EMT and a stem-like phenotype and stimulated integrin signaling through FAK kinase phosphorylation. This resulted in accelerated tumor growth and possible resistance to radiation therapy (Katakam et al., 2020). Similarly, in gastric carcinomas, high Sdc-1 expression correlated with a less aggressive tumor phenotype (Charchanti et al., 2019). Notably, normal tissues of the gastro-intestinal tract have high levels of Sdc-1 (Schick et al., 2001), which suggests that Sdc-1 expression is lost at some point during cancer development and progression in some of the patients, conferring a survival advantage.

This is supported by several studies, which show that Sdc-1 expression is lost during EMT in both cancer (Yang et al., 2011; Farfán et al., 2018; Kalscheuer et al., 2019) and normal tissues (Sun et al., 1998). EMT is an important developmental program of embryogenesis, which can be hijacked by the cancer cells during tumor growth to promote invasion and metastasis. As previously mentioned, increased tissue stiffness promotes EMT and up-regulates mechanosignaling in cancer (Wei et al., 2015; Zhang et al., 2016; Matte et al., 2019). This raises a possibility that high tumor stiffness promotes a mesenchymal-like phenotype in cancer cells, which in turn leads to the loss of Sdc-1, among other consequences.

#### Syndecan 2, NG2, Betaglycan

Several other transmembrane proteoglycans have also been linked to an aggressive cancer phenotype. Syndecan 2 (Sdc-2) expression is increased in a range of cancers including lung adenocarcinoma and colon cancer, where it promotes expression of matrix metalo-proteinase 9 and 7 (MMP9 and MMP7) respectively (Jang et al., 2016; Tsoyi et al., 2019). MMPs regulate cancer cell invasion by digesting the ECM immediately surrounding the cancer cells, and by inducing shedding of E-cadherin adhesion proteins. Sdc-2 is therefore linked to the EMT and cancer invasion (Mytilinaiou et al., 2017). Similarly, Syndecan 4 (Sdc-4) has been shown to promote EMT in the papillary thyroid carcinoma cells (Chen L.L. et al., 2018). NG2 is another transmembrane proteoglycan, which is often over-expressed in cancer and has been linked to poor prognosis (reviewed in Nicolosi et al., 2015).

In contrast to the transmembrane proteoglycans discussed above, betaglycan, also known as TGFβ-receptor 3 (TGFβR3), has been associated with positive outcome in cancer patients (Nicolosi et al., 2015). Nicolosi et al. found that abundant betaglycan inhibits NF-κB and MAPK signaling and reduces tumor growth and invasion. In line with this, the loss of betaglycan in renal cell carcinoma lifts this inhibition and induces TGF-β signaling. This results in rapid metastasis *in vivo* (Nishida et al., 2018).

### Transmembrane Glycosyl-Phosphatidyl-Inositol (GPI) – Anchored Proteoglycans

#### Glypicans

A recent study described activation of Wnt/β-catenin/c-Myc signaling axis by one of the transmembrane GPI-anchored proteoglycan glypican-4 (GPC4), and the resulting up-regulation of glycolysis in colorectal cancer. Moreover, proteasomal degradation of GPC4 resulted in Wnt/β-catenin/c-Myc signaling attenuation and metabolic reprogramming, which was favorable for the survival of the tumor-bearing mice (Fang et al., 2019). By contrast, glypican-3 (GPC3) has been reported to act as a tumor suppressor. GPC3 silencing promoted breast cancer growth, while ectopic re-expression of GPC3 inhibited growth *in vitro* (Xiang et al., 2001).

#### Neuropilin-1

The transmembrane proteoglycan Neuropilin-1 (NRP1) is one of the proteoglycans which serves as a co-receptor in several growth-factor signaling pathways, including EGFR-Akt (Rizzolio et al., 2012), fibroblast growth factor 2 (FGF2) (West et al., 2005), PDGF – TGFβ (Cao et al., 2010) and VEGF signaling. Notably, NRP1-mediated activation of the VEGF signaling is dependent on the HS or CS GAG chain attachment to its core protein. In the absence of full glycosylation, stimulation with VEGF no longer promotes growth, invasion and formation of blood vessels (Shintani et al., 2006; Hendricks et al., 2016).

### Pericellular and Basement Membrane – Anchored Proteoglycans

#### Perlecan

Perlecan is a proteoglycan highly expressed in basement membranes, peri-vascular regions and tumor-adjacent stroma regions in most solid cancer types (Figure 3) (Cruz et al., 2020). Multiple HS chains attached to perlecan protein core account for its ability to bind a variety of growth factors, cytokines and cell surface receptors (Whitelock et al., 2008). Controlled gradual release of these bound growth factors maintains structured tissue homeostasis, which becomes impaired in malignant tumors (Cruz et al., 2020).

Perlecan has been identified in a recent study, as a mediator of the intricate relationship between cancer cells and CAFs in PDAC (Vennin et al., 2019). The authors found that PDAC cells carrying a p53-gain of function (GOF) mutation, induced a transcriptional change in CAFs, prompting them to support invasion and metastasis. In these tumors, CAFs stimulated cross-linking of collagen fibers, which resulted in higher mechanical stiffness levels compared to the less aggressive counterparts with the loss of p53. Notably, conditioned media, collected from CAFs of p-53-GOF tumors was sufficient to induce collagen remodeling *in vitro*, suggesting that some of the soluble secreted factors are responsible for this change. A mass spectrometry analysis of the CAF-derived secretome revealed a high abundance of perlecan. Further experiments demonstrated that perlecan was necessary and sufficient for induction of collagen remodeling, increase in cancer cell actomyosin contractility and promotion of invasion of the PDAC cells into the matrix (Vennin et al., 2019). Moreover, immune response against the tumor cells was significantly increased following perlecan depletion. In line with this, antibody targeting perlecan inhibited tumor growth in a PDX mouse model of TNBC. Interestingly, a stronger anti-tumor effect was observed in nude mice than in the Nod/scid (NSG) mice, suggesting that perlecan helps create an immunosuppressive tumor microenvironment (Kalscheuer et al., 2019).

It has also been reported that during bone metastasis in prostate cancer, accumulation of perlecan promotes cell-cell adhesion instead of cell-ECM, preventing spreading of the cancer cells. Perlecan cleavage by matrix metalloproteinase-7 (MMP-7) facilitates cancer cell-ECM adhesion, dispersion and eventually leads to bone metastasis via induction of FAK-dependent invasion (Grindel et al., 2018). These examples underscore the highly context-dependent manner in which various proteoglycans impact tumor progression.

#### Agrin

In addition to perlecan, other pericellular proteoglycans have also been shown to have pro-metastatic functions. Agrin, another HS-bearing proteoglycan, has a limited expression in the normal tissue, but is progressively up-regulated during malignant transformation in oral cancer (Rivera et al., 2018). A knock-down of agrin reduced cancer cell proliferation, invasion and sphere formation. Similarly to perlecan, agrin from conditioned media was sufficient to rescue proliferation and invasion in the agrin-silenced cells, suggesting that soluble agrin mediates its pro-metastatic function. In addition, it has been shown that agrin facilitates angiogenesis. Agrin produced by cancer cells, binds to and stabilizes its receptors β1-integrin and Lrp4 on the surface of the vascular endothelial cells. Coupled with an increasing stiffness inside a tumor, this interaction promotes formation of focal adhesions and stabilization of VEGFR2. Activation of VEGFR2, in turn, leads to branching and proliferation of the endothelial cells, and formation of new blood vessels (Njah et al., 2019).

#### Collagen XVIII and Collagen XV

Other pericellular and basal membrane proteoglycans, such as collagen XVIII and collagen XV have also been implicated in cancer progression support, however, limited evidence has been reported on this subject. In metastatic gastric cancer, as well as in non-small cell lung cancer, high expression of collagen XVIII correlated with a poor clinical outcome (Iizasa et al., 2004; Lee et al., 2010). However, both collagen XVIII and XV are integral components of the basal membrane. While their expression in the tumor may be up-regulated, both collagens are often depleted from the basement membrane. This makes it more permeable and thus enables epithelial tumor cells to invade (Amenta et al., 2003).

### Extracellular

Abundance of extracellular proteoglycans in the ECM has been linked to a more aggressive phenotype of the tumors and stiffer microenvironment (Varga et al., 2012). Accumulation of ECM proteoglycans is also known to result in higher tissue stiffness. In a normal breast, higher tissue stiffness corresponds to higher mammographic density (MD), a well-established risk-factor for breast cancer development, as mentioned above (Vachon et al., 2007). High MD is characteristic for breast tissue rich in ECM proteoglycans, including hyalectans (aggrecan, versican, neurocan, and brevican), basement membrane proteoglycans (ex. perlecan), and SLRPs such as lumican and decorin (Alowami et al., 2003; Shawky et al., 2015).

It is important to note, however, that while proteoglycan expression level is often associated with cancer, the extent of their modification with GAG chains may be even more important. A recent study examined a pattern of extracellular proteoglycans in the ECM of malignant brain tumors. The authors discovered that CS-modified proteoglycans, such as aggrecan, versican, neurocan, and brevican, concentrated on the edge of the low-grade gliomas. The CS GAGs promoted adhesion between tumor cells and attracted reactive astrocytes, thus helping limit the invasion of the tumor cells into the brain tissue. The more aggressive high-grade tumors, however, had a low abundance of CS GAGs, which the authors connected to the invasive nature of those lesions (Silver et al., 2013). This proposed barrier function is supported by evidence from previous reports, where extracellular proteoglycans were found to direct brain tissue development by imposing spatial limits between distinct regions of the nervous tissue and the outer borders of the brain and spinal cord (Golding et al., 1999).

#### Testicans

Testican 1, encoded by SPOCK1 gene, has been shown to promote cancer growth. SPOCK1 knock-down strongly reduces tumor growth and metastasis in an *in vivo* model of breast cancer (Perurena et al., 2017). Similarly, in colorectal cancer, SPOCK1 was found to be over-expressed and contributed to the activation of PI3K-Akt signaling (Zhao et al., 2016). This is in agreement with a recent report that demonstrated activation of the same signaling cascade in glioma cells (Yang et al., 2016). In all cases, tumor-supporting role of testican 1 was linked to induction of EMT and activation of Wnt signaling (Miao et al., 2013). A link between testican 1 expression and EMT is further supported by evidence that resistance to a broad range of targeted inhibitors, associated with a mesenchymal-like phenotype, is reversed upon testican 1 knock-down (Kim et al., 2014).

The role of other members of the testican family is less clear, as there are only a handful of published reports on this subject. It appears that testicans have a role in the inhibition of MMPs, a group of enzymes that catalyze matrix digestion and facilitate tumor cell invasion. Testican 3 was found to inhibit certain MMPs, potentially preventing cancer spread. Conversely, testican 2 was found to have an opposite effect, counteracting the inhibition imposed by testican 3 (Kim et al., 2014). In summary, contribution of the broader testican family to cancer progression requires further investigation.

#### Small Leucine-Rich Proteoglycans (SLRPs)

The role of SLRP family of proteoglycans, which includes decorin, biglycan and lumican, in cancer is different between its members (reviewed in Appunni et al., 2019). SLRPs are abundant in the ECM of most tissues, where they bind to various cell receptors, regulating signaling cascades. For example, one of the SLRPs, decorin, has been shown to bind multiple receptor-tyrosine kinases, inhibiting their activation (Neill et al., 2016). Decorin was shown to have a particularly high affinity for the EGFR. Binding of decorin to EGFR led to ERK-mediated expression of p21 and subsequent cell cycle arrest (Moscatello et al., 1998). In addition, decorin can engage toll-like receptors 2 and 4, inducing inflammatory response. These functions explain the tumor suppressive and anti-metastatic role of decorin, which has been reported across several cancer types (reviewed in Neill et al., 2016).

#### Hyalectins

Some of the members of the hyalectin family of proteoglycans have been shown to play a role in cancer. For instance, elevated levels of versican have been reported in gastric (Shen et al., 2015), lung (Asano et al., 2017), and endometrial cancer (Kodama et al., 2007), where its expression correlated with poor clinical prognosis. In breast cancer, expression of versican was only enriched in malignant tumors, but not in the non-malignant lesions (Vachon et al., 2007).

Brevican, has been found to promote motility of astrocytoma cells, supporting progression of this cancer from low- to high-grade glioma (Lu et al., 2012). Neurocan, another hyalectan that is found in the CNS, is increased in neuroblastomas. Several studies found that high neurocan expression has a strong correlation with shortened patient survival (Su et al., 2017; Zhong et al., 2018).

#### Hyaluronan

Hyaluronan, also referred to as hyaluronic acid, is a unique GAG which is composed entirely of the non-sulfated repeating disaccharides made of *N*-acetyl glucosamine (GlcNAc) and D-GlcA residues. As it does not have a protein core, it cannot be considered a proteoglycan. However, hyaluronan binds to multiple proteins and proteoglycans within the ECM and contributes greatly to structural integrity of different tissues, as well as to cancer development and progression. Its role in cancer is diverse and has been a subject of several comprehensive reviews (Rankin and Frankel, 2016; Liu et al., 2019).

## Proteoglycans Are Involved in Mechanosignaling

### ECM Proteoglycans

Stiffer tumors are characterized by two important changes involving proteoglycans: a bulkier glycocalyx of the cancer cells, and a stiffer, denser ECM. Both of these changes require abundant proteoglycans with long, bulky GAG chain modifications (Table 1). Proteoglycans and hyaluronan, in either unbound or bound form, make up a significant part of cancer ECM and are actively involved in the regulation of its stiffness. One of the starkest pieces of evidence to support this comes from an unusual source – a study of the human tooth. The authors describe the proteoglycans present at the interfaces between different regions of the tooth that come under high levels of mechanical stress. These KS- and CS-decorated SLRPs are essential for the structural integrity of the tooth, as their enzymatic digestion reduces the stiffness of the tissue, making it less resistant to mechanical loads (Kurylo et al., 2016). The implication of this finding is that proteoglycans can markedly contribute to the regulation of tissue mechanics.

**TABLE 1:** Proteoglycans control mechanical properties of the tissue and regulate mechanosignaling.

**PGs regulate ECM stiffness**		
Biglycan and fibromodulin	Maintain structural integrity and load-bearing capacity in the human periodontal complex	Kurylo et al., 2016
Biglycan and decorin	Control collagen fiber assembly	Iozzo et al., 1999; Zhang et al., 2006; Lewis et al., 2010; Robinson et al., 2017
Biglycan	Controls collagen crosslinking and fiber density	Andrlová et al., 2017
Agrin	Stabilizes focal adhesion complexes, promotes ECM stiffness and mechanosignaling	Chakraborty et al., 2015, 2017; Njah et al., 2019
Aggrecan	Prevents ECM stiffening in cartilage tissues	Nia et al., 2015
**PGs modulate glycocalyx bulk and tune mechanosignaling**		
Sdc-1	Promotes β1-integrin signaling, invasion, cell spreading and re-alignment in response to shear force	Iba et al., 2000; Beauvais and Rapraeger, 2010; Sarrazin et al., 2011; Vuoriluoto et al., 2011; Yang et al., 2011; Ebong et al., 2014
Sdc-4	Conveys direct mechanical cues to β1-integrins, binds ECM protein, supports adhesion complexes and mechanosignaling	Woods and Couchman, 2001; Okina et al., 2012; Cavalheiro et al., 2017; Kennelly et al., 2019; Chronopoulos et al., 2020
Syndecans	Interact with PDZ-domain proteins, control cytoskeletal remodeling, cell spreading and migration	Grootjans et al., 1997; Hsueh et al., 1998; Tkachenko et al., 2006; Sulka et al., 2009; Kashyap et al., 2015; Keller-Pinter et al., 2017
**Serglycin**		
Serglycin	Promotes FAK signaling and mechanosignaling	Zhang et al., 2020

*Notable examples outlined in this review.*

In cancer, perhaps the most notable example of this are the tumors of the CNS. Brain ECM contains large quantities of hyaluronan (Wolf et al., 2019), CS-modified proteoglycans, predominantly lecticans, as well as small glycoproteins and adhesive molecules – laminin, tenascin C, and fibronectin. Proteoglycans interact with the adhesive molecules, creating a network of varying density. For example, hyaluronan binding to tenascin C results in a more rigid ECM with higher stiffness. This is coupled with the fact that tenascin C is over-expressed in malignant tumors and is distributed in a gradient manner, with the highest abundance in the central regions, decreasing toward the periphery. This was shown to be controlled by HIF1α (Miroshnikova et al., 2016; Chen J.E. et al., 2018), which is highly expressed in the poorly vascularized, hypoxic regions in the core parts of the tumors. Both the presence of hypoxic areas and high stiffness have a strong association with poor prognosis in glioblastomas, suggesting that hypoxia-driven production of tenascin C, and its binding to hyaluronan underlie the mechanism of stiffness increase, which fuels glioblastoma progression (Miroshnikova et al., 2016). Other studies have shown that ECM enrichment with hyaluronan independently induces a stretched, contractile phenotype in glioma cells, promoting their invasion (Wang et al., 2014; Pogoda et al., 2017). Notably, hyaluronan abundance does not induce tumor cell proliferation, but instead reduces tumor volume. Despite this, it is associated with poor prognosis, as abundant hyaluronan promotes cancer cell invasion and dissemination, rather than the growth of a single parental tumor.

Outside of the CNS, proteoglycans are involved in mechano-transduction by modulating ECM composition and organization. Two SLRPs – decorin and biglycan are known to be essential for the regulation of collagen fiber assembly in tendons. A knock-down of these proteoglycans results in larger collagen fibers that can tolerate smaller mechanical loads. The overall tendon stiffness is also reduced upon decorin and biglycan knock-down (Zhang et al., 2006; Robinson et al., 2017). In a follow-up study, it was determined that the DS GAG chains attached to decorin were required for binding and remodeling of collagen (Lewis et al., 2010). These and other studies link decorin to mechano-regulation and indicate that the presence of SLRPs is important for the maintenance of soft ECM environments. In support of this, decorin acts as a tumor suppressor in many cancer types, while its loss precipitates cancer progression (Iozzo et al., 1999). Another SLRP, biglycan also contributes to the ECM remodeling. In melanoma models, the loss of biglycan expression in mouse embryonic fibroblasts results in softer ECM with less collagen crosslinking and reduced collagen fiber density. Among patients with melanoma, low expression of biglycan, either by the tumor or the stroma cells, is strongly linked to better clinical prognosis (Andrlová et al., 2017). In summary, the evidence of SLRPs’ involvement in regulation of mechanosignaling in cancers is still limited and merits further investigation.

#### Agrin

Mounting evidence points to another secreted proteoglycan – agrin as a modulator of mechanosignaling in cancer. Agrin was first described as a ligand of Lrp4, which binds to a scaffolding protein MUSK. This complex serves as a platform for multiple signaling pathways, supporting cancer growth and preventing apoptosis (Zong and Jin, 2013). One of the down-stream signaling cascades that is activated by agrin is YAP/TAZ (Chakraborty et al., 2017).

In the same study, the authors uncovered the mechanism of mechano-sensing by agrin. Cells cultured on stiff substrates produce and secrete higher amounts of agrin. In turn, abundant agrin in the ECM potently increases stiffness of the tumors *in vivo*, which is dependent on collagen accumulation. Through its receptors, agrin stimulates YAP signaling, inducing YAP nuclear localization. Conversely, a knock-down of agrin results in YAP translocation back into the cytosol and a decreased expression of YAP target genes. Notably, this effect is exclusively mediated through agrin, while stiffness alone in absence of agrin expression is not sufficient to activate YAP and its target genes (Chakraborty et al., 2017). Corroborating the role of agrin in mechanosignaling, in the subsequent study of hepato-carcinomas, it was determined that agrin stabilizes focal adhesion complexes and facilitates FAK signaling. As a consequence, agrin depletion impedes cell adhesion and invasion and has a dramatic effect on tumor growth, practically eliminating tumors completely (Chakraborty et al., 2015).

In support of these findings, it was shown that agrin, produced both by the cancer cells, as well as the vascular endothelial cells, is required for activation of angiogenesis, blood vessel sprouting and cancer cell adhesion to the endothelial cells (Njah et al., 2019). The authors found that agrin binding to its Lrp4 receptor, induced β1 integrin – FAK signaling, formation of focal adhesion complexes and stabilized protein levels of VEGFR2, which is critical for endothelial cell migration and proliferation. Importantly, these agrin-mediated effects were stiffness-dependent, suggesting that stiff environments in tumors promote angiogenesis via agrin-mediated mechanosignaling (Njah et al., 2019).

#### Aggrecan

Aggrecan is another proteoglycan of the ECM, whose expression is primarily limited to cartilage. Its abundance and modifications determine cartilage stiffness and elasticity. It has been shown that the length of CS and KS GAG chains attached to aggrecan decreases with age, along with an overall depletion of aggrecan. This results in progressively stiffer tissue (Nia et al., 2015). While this change occurs in normal development, it may become exacerbated with age and lead to development of osteoarthritis – degradation of cartilage (Alberton et al., 2019). It is feasible that aggrecan expression and the length of its GAG chains contribute to the regulation of osteosarcomas, or metastasis of other cancers to the bone. However, to the best of our knowledge, this has not yet been investigated.

### Cell Surface Proteoglycans

**FIGURE 4:**
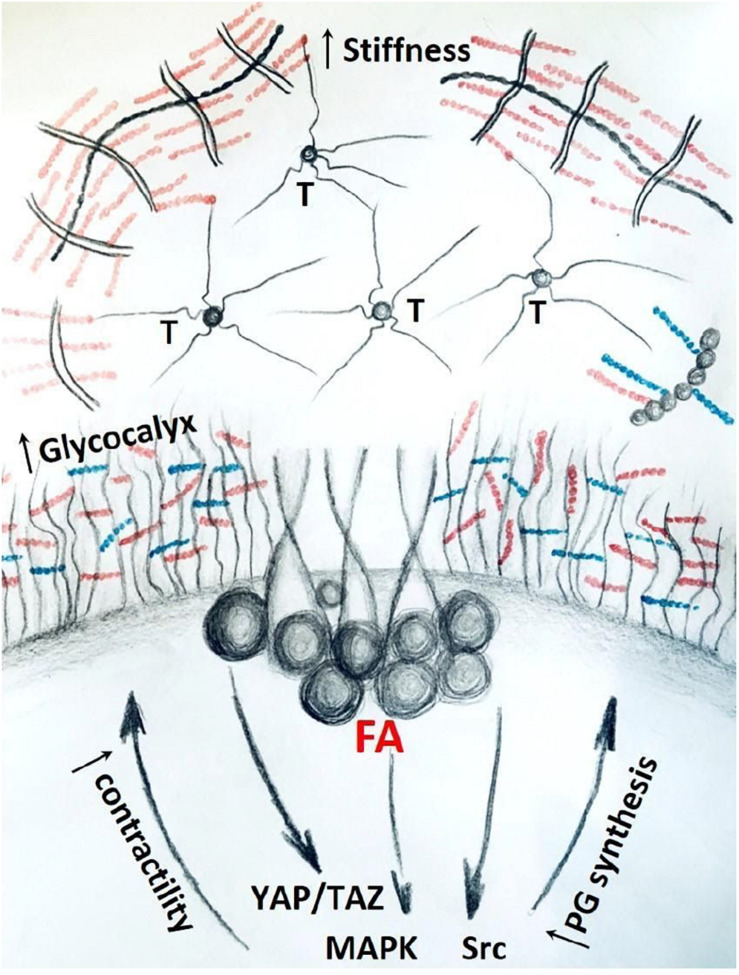
Stiff matrix, rich in tenascin (T) and proteoglycans promote bulkier glycocalyx and up-regulate mechanosignaling through focal adhesions (FA) and down-stream pathways. Activation of mechanosignaling results in production of more proteoglycans that contribute to bulkier glycocalyx and stiffer ECM, thus concluding a positive-feedback loop in tumor tissue.

The role of ECM proteoglycans in mechanosignaling has not been fully investigated. However, more is known about the transmembrane and peri-cellular proteoglycans in the context of mechano-regulation. It has been reported that tumors with up-regulated tissue mechanics have bulkier glycocalyx (Barnes et al., 2018). In addition, bulky glycocalyx further amplifies mechanosignaling through focal adhesions, creating a positive-feedback loop which propagates malignant phenotype (Figure 4) (Woods et al., 2017). As key components of glycocalyx, proteoglycans at the interface of the cell-to-matrix interaction, contribute to the regulation of this response.

#### Syndecans

Some proteoglycans can serve as co-receptors of integrins. One of the best-studied examples of that is syndecans, which facilitate recognition and binding of external ligands, including growth factors and components of the ECM, through their HS chains within the range of (≈300 nm (Sarrazin et al., 2011). Syndecans interact with different integrins on the cell surface, forming diverse combinations. Their binding can occur both within the cytoplasmic and the extracellular domains, and facilitates formation and maturation of the adhesion complexes. Examples of this include interaction of Sdc-4 and Sdc-1 with α2β1 integrins, which promotes invasion of KRAS-mutant cells into collagen gels (Vuoriluoto et al., 2011). In addition, it was found that Sdc-1 mediates coupling of αvβ3 integrin with IGFR1 (Beauvais and Rapraeger, 2010). Sdc-1 directly binds to the integrins, which prompts formation of an integrin-IGFR1 complex and IGFR1 auto-phosphorylation. Integrin interaction with IGFR1 was found to be necessary for the activation of integrin signaling and cell spreading on substrates (Beauvais and Rapraeger, 2010).

Sdc-1 binding to disintegrin and MMP domain-containing protein 12 (ADAM 12) induces a conformational change that promotes binding of *β*1 integrins and subsequent cell spreading (Iba et al., 2000). In line with this, Sdc-1 expression in stromal fibroblasts induces rearrangement of the collagen fibers, allowing breast cancer cells to adhere and extravasate more efficiently (Yang et al., 2011). In summary, these studies provide evidence that Sdc-1 is involved in focal adhesion formation and supports activation of the down-stream signaling.

In addition, cell surface proteoglycans have been shown to act as mechano-sensors of shear force in vascular endothelial cells. In normal conditions, the endothelial cells align themselves with the direction of the flow, creating a uniform layer. One study has found that debulking of the endothelial glycocalyx with HS degrading heparinase III prevents such re-alignment of the cells. The authors found that this effect is dependent on Sdc-1, as the Sdc-1 knock-down also abolished cell realignment as well as remodeling of the fibrillar actin network, while a knock-down of glypican-1 did not (Ebong et al., 2014). The distinct roles of these proteoglycans in shear force sensing may be explained by the difference in their membrane tethering. Sdc-1 transverses the plasma membrane with a transmembrane domain that serves as a stable anchor connecting it to the cytoskeleton. In contrast, glypican-1 is more loosely attached with a GPI anchor and is often found within lipid rafts in the membrane. A recent study found that shear stress conditions induce re-distribution and clustering of glypican-1 on the cell surface, while Sdc-1 remains in place (Zeng et al., 2013).

Sdc-4 has also been described as essential for the formation and stabilization of focal adhesions (Woods and Couchman, 2001). Evidence in support of this includes a study, where mechanical force applied directly to the Sdc-4 through magnetic pulling of the conjugated beads, resulted in activation of β1-integrins, activation of down-stream signaling and an increase in cortical stiffness (Chronopoulos et al., 2020). Conversely, cells with a knock-down of Sdc-4 lose FAK and *β*1 integrins expression at the leading edge and fail to establish stable adhesions. Sdc-4 silencing also results in un-coupling of vinculin from filamentous actin network and its dispersion in the cytosol. As a result of these events, loss of Sdc-4 leads to reduced adhesion and increased proliferation (Cavalheiro et al., 2017). Furthermore, Sdc-4 has been shown to bind to the ECM proteins, such as fibronectin, with the HS chains, forming a stable deformation-resistant bond, adding to the strength of integrin-fibronectin binding (Kennelly et al., 2019).

Sdc-4, has been shown to directly interact with an α-actinin. α-actinin is an actin fiber-remodeling protein, which cooperates with myosin II to facilitate cell contractility and locomotion (Figure 2). Sdc-4 therefore serves as an important sensor of mechano-stimulation and regulator of cellular traction force (Okina et al., 2012).

All syndecans have also been shown to interact with a diverse group of PDZ-domain carrying proteins, which are important for tethering receptor proteins to the plasma membrane and serve as scaffolding proteins in signaling cascades (Cheng et al., 2016). Syndecans interact with PDZ-domain proteins through a conserved tetra-peptide region of their intracellular domain (Grootjans et al., 1997; Hsueh et al., 1998). These syndecan-PDZ protein complexes have been shown to be important for the cytoskeletal rearrangement, cell spreading and migration, with implications for cancer dissemination and metastasis (Tkachenko et al., 2006; Kashyap et al., 2015). Since there are four different syndecans and over 250 PDZ-domain proteins, specificity of their interactions is regulated through post-translational modifications of both the PDZ-domain and the cytoplasmic domain of syndecans, within or next to the PDZ-binding region. For example, phosphorylation of the cytoplasmic region of Sdc-1 prevents its interaction with syntenin-1 (Sulka et al., 2009), but it strengthens its binding with T-lymphoma invasion and metastasis-inducing protein 1 (Tiam1) (Keller-Pinter et al., 2017) – both PDZ-domain proteins, which regulate cytoskeletal organization and cell migration.

### Serglycin in Mechanosignaling

Finally, serglycin, as the only known intracellular proteoglycan has not been thoroughly investigated in terms of its contribution to mechanosignaling. One report has linked high abundance of serglycin with up-regulation of both mRNA expression and nuclear localization of YAP/TAZ (Zhang et al., 2020). YAP/TAZ activation is known to occur in response to multiple different inputs, although the mechanism of serglycin-mediated activation of YAP/TAZ is not completely understood. Stimulation of cells with either serglycin over-expression, or treatment with conditioned media collected from serglycin over-expressing cells, induced FAK signaling. This suggests that secreted serglycin promotes focal adhesion signaling, which in turn activates YAP/TAZ. In line with this, serglycin knock-down prevented activation of YAP/TAZ and the down-stream genes in a breast cancer model. This resulted in improved sensitivity to chemo-therapeutic treatment (Zhang et al., 2020).

Considering this intriguing observation, further studies are warranted to better understand the regulation of mechanosignaling by serglycin in cancer cells.

## Proteoglycans in Cancer Therapy and Diagnosis

As outlined in chapters above, proteoglycans contribute to cancer progression, which is at least partially mediated through their role in mechanosignaling. From this perspective, proteoglycans represent attractive potential therapeutic targets. The search for clinical applications began in 1983 with the first report of the anti-angiogenic and anti-tumor properties of unfractionated heparin (Folkman et al., 1983). Since then, cancer type- and tumor stage-specific expression of certain proteoglycans have been described, positioning them as useful diagnostic and prognostic markers. For example, high expression of pericellular agrin on the interface between the cancer cells and the surrounding ECM, serves as a strong negative prognostic marker in PDAC. Knock-down of agrin, on the contrary, reduces both the primary and the metastatic tumor load *in vivo* (Tian et al., 2020). The study points to agrin’s role in the modulation of mechanosignaling, and support of adhesion and migration as the likely explanation for this effect. Changes in proteoglycan abundance and modifications also contribute to the ECM remodeling during tumor growth and the development of therapeutic resistance (Theocharis and Karamanos, 2019). For example, in melanoma, resistance against BRAF-inhibitor was accompanied by increased ECM levels of versican and biglycan (Girard et al., 2020). There is therefore evidence that inhibition of mechanosignaling, regulated by certain proteoglycans holds substantial therapeutic promise, although it mostly remains to be explored.

Another avenue of modifying proteoglycans for therapeutic gain is through modulation of their enzymatic processing (Shen et al., 2015). GAG chains attached to the protein cores of proteoglycans can be modified by sulfatases, which remove a sulfo group on HS chains, reducing the binding of growth factors. GAG chains can also be shortened or cleaved with a similar effect by appropriate enzymes, depending on the composition of the chain. For instance, in pancreatic cancer, increased levels of heparanase – the enzyme that cleaves HS GAGs, have been implicated in angiogenesis and metastasis, and correlated with poor prognosis (Pirinen et al., 2005).

This prompted investigation of heparanase inhibitors as potential therapeutics. For example, a significant tumor cell growth reduction *in vitro* and prolonged survival *in vivo* were reported upon treatment with a HS-mimetic heparanase inhibitor PG545. In addition to the survival advantage, PGP545 treatment also changed tumor cell phenotype, with the remaining tumor showing reduced levels of VEGF, vimentin, and collagen I. Histological analysis of these tumors showed high level of differentiation, suggesting that PGP545 may reverse tumor EMT (Ostapoff et al., 2013). In agreement with these findings, other studies found that PG545 treatment inhibited angiogenesis and reduced tumor burden in mouse models of breast, lung and prostate cancer (Kodama et al., 2007). Despite these promising preclinical results, as of 2019, no heparanase inhibitor has been tested in patients, as selective targeting of heparanase remains challenging (Shen et al., 2015).

Additionally, expression of specific proteoglycans by the tumor tissues can be exploited in various targeted drug delivery systems (Fuster and Esko, 2005; Misra et al., 2015). Nanocarrier systems such as liposomes or antibody-drug conjugates can improve drug delivery and anti-tumor activity of classical chemotherapeutics, while reducing their toxicity (Peer et al., 2007). Lee et al. developed cisplatin-loaded CS-binding liposomes, that effectively inhibited tumor growth in liver metastasis murine model (Lee et al., 2002). Similarly, antibody-drug conjugate targeting CSPG4 triggered melanoma regression *in vivo* (Hoffmann et al., 2020). Several similar approaches that utilize HSPGs have been proposed for treatment of breast (Khatun et al., 2013) and liver cancers (Longmuir et al., 2009).

Several groups identified cell surface proteoglycan glypican-1 as a specific marker of circulating cancer-cell-derived exosomes in pancreas and breast tumors. Glypican-1-positive exosomes may therefore serve as a tool for early detection of cancer (Melo et al., 2015). Similarly, HS proteoglycan Sdc-1 has been reported as an exosome constituent specific for glioblastoma, but not low-grade glioma (Indira Chandran et al., 2019).

Recently, chimeric antigen receptor (CAR) T-cells have been developed as a therapeutic tool that showed an impressive efficacy in B-cell lymphomas (Jackson et al., 2016). Subsequently, several cell surface proteoglycans have been suggested as CAR T-cell targets in solid tumors. Pellegatta et al. reported CART treatment targeted at CS proteoglycan 4 (CSPG4) was effective in glioblastoma mouse models (Pellegatta et al., 2018). Similarly, Glypican-3 was suggested as CAR-T-cell target for melanoma, lung and hepatocellular carcinoma, respectively (Shimizu et al., 2019). Despite groundbreaking discovery of CAR T-cell therapy in hematopoietic malignancies, their application in treatment of solid tumors remains challenging, likely due to the characteristic microenvironment of solid tumors (Newick et al., 2017). In summary, application of proteoglycans in cancer therapy holds potential, but requires further studies.

## Conclusion and Future Perspectives

The role of tissue mechanics and mechanosignaling in tumor development and progression is slowly becoming appreciated. Considering that proteoglycans make up a significant part of the ECM and serve as an interface between the cancer cells and their environment, it is safe to assume their involvement in the regulation of mechano-sensing and mechanosignaling. In this review, we summarize published reports that support this assumption. We conclude that the role of proteoglycans in mechanosignaling deserves thorough investigation, as it may uncover novel therapeutic approaches that could potentially curb cancer progression in patients.

## Author Contributions

AB and VMW both conceived and wrote the review. ABJr and MŽ contributed sections of the review. All authors contributed to the article and approved the submitted version.

## Conflict of Interest

The authors declare that the research was conducted in the absence of any commercial or financial relationships that could be construed as a potential conflict of interest.

## Abbreviations

bFGF: basic fibroblast growth factor
CSC: cancer stem cell
CAFs: cancer-associated fibroblasts
CNS: central nervous system
CS: chondroitin sulfate
JNK: c-JunN-terminal kinase
DS: dermatan sulfate
EGFR: epidermal growth factor receptor
EMT: epithelial to mesenchymal transition
ER: estrogen receptor
ECM: extracellular matrix
FGF: fibroblast growth factor
FAK: focal adhesion kinase
FA: focal adhesion
GlcA: glucuronic acid
GPI: glycosyl-phosphatidyl-inositol
GPC3, GPC4: glypican-3, 4
GAG: glycosaminoglycan
HS: heparan sulfate
HSPG: heparan sulphate proteoglycans
HIF1 α: hypoxia-inducible factor 1 α
IdoA: iduronic acid
IGFR1: insulin-like growth factor receptor 1
KS: keratan sulfate
Lrp4: lipoprotein-related receptor-4
MAPK: MAP kinase
MMPs: matrix metalloproteinases
MUSK: MMP domain-containing muscle-specific kinase
GalNAc: *N*-acetylgalactosamine
GlcNAc: *N*-acetylglucosamine
NCAMs: neural cell adhesion molecules
NG2: Neural/glial antigen 2
NRP1: Neuropilin-1
PDAC: pancreatic ductal adenocarcinoma
TAZ: PDZ-binding motif
ROCKI and ROCKII: Rho-associated protein kinase 1 and 2
SLRPs: small leucine-rich proteoglycans
Sdc-1, 2: Syndecan 1 and 2
TGF β R3: transforming growth factor β-receptor 3
VEGFR: vascular endothelial growth factor receptor
VASP: vasodilator-stimulated phosphoprotein
YAP: yes-associated protein
ADAM 12: protein 12.
